# Pericardium-Focused Osteopathic Treatment in Chronic Cervicothoracic Pain Following Median Sternotomy for Cardiac Surgery

**DOI:** 10.7759/cureus.103962

**Published:** 2026-02-20

**Authors:** Harbi Shehadeh

**Affiliations:** 1 Osteopathy, Private Practice in Physiotherapy and Osteopathy, Madrid, ESP

**Keywords:** chronic cervicothoracic pain, fascial restriction, median sternotomy, osteopathic manipulative treatment, pericardium

## Abstract

Chronic cervicothoracic pain may develop following median sternotomy, with an underlying pathophysiology that remains incompletely elucidated. Pericardial fascial restriction and mediastinal adhesions have been proposed as potential contributors to the onset and persistence of symptoms. We report the case of a 72-year-old man with a history of triple coronary artery bypass grafting who presented with chronic cervicothoracic pain and restricted cervical rotation. An osteopathic manipulative treatment (OMT) protocol focused exclusively on the pericardium was applied, comprising four sustained-tension techniques delivered across three sessions. Pre- and post-intervention assessments were conducted at each session, in addition to a final evaluation 15 days after the last session (day 35) and a further telephone follow-up 32 days later (day 67), without objective reassessment at this final contact. Objective improvements were observed in cervical rotation measured using digital inclinometry (right/left: 34°/38° to 54°/55°) and in pressure pain threshold assessed by algometry over C7 (1.2-2.8 kg/cm^2^), together with a reduction in pain intensity, as measured by the numeric pain rating scale (NPRS), from 7 to 2. No adverse events were reported. Although these findings should be interpreted with caution, given the single-case design, they suggest that a pericardial-focused approach may be associated with clinically meaningful improvements in cervical mobility and chronic post-sternotomy cervicothoracic pain. Controlled studies with larger sample sizes, standardised outcome measures, and assessor blinding are required to evaluate its efficacy and to further elucidate the underlying neurophysiological mechanisms involved.

## Introduction

Following cardiac surgery, particularly when performed via median sternotomy, the development of persistent thoracic pain has been described as a clinically relevant complication [1-5]. In addition to localised sternal pain, some cases reported in the literature have described associated cervicothoracic symptoms or radiation to anatomical structures related to the mediastinum [1,5]. However, the available evidence regarding this specific presentation remains limited and is primarily based on case reports and case series.

Chronic postsurgical pain is defined as pain that develops or increases following a surgical procedure and persists beyond the normal healing process, that is, for at least three months after the initial event, excluding other causes, such as infection, malignancy, or pre-existing pain [6]. Estimates of the incidence of chronic post-sternotomy pain following cardiac surgery vary widely across the literature, depending on study design, type of surgical procedure, and duration of follow-up. In prospective studies specifically addressing chronic post-sternotomy pain, prevalence rates of approximately 11% at one year after surgery have been reported, with a progressive decline to around 3-4% at five years of follow-up, reflecting spontaneous resolution in a substantial proportion of initially symptomatic patients [3]. The literature also reflects considerable heterogeneity in the reported figures, attributable to methodological differences and the diagnostic criteria employed [6].

The pathophysiology of chronic pain secondary to median sternotomy remains insufficiently elucidated. Various mechanisms have been proposed to account for its development, including surgically induced tissue trauma, potential involvement of the intercostal nerves during internal mammary artery dissection, nerve entrapment phenomena, and mediastinal scar-related alterations [5]. In the postoperative context, pain following sternotomy has been described as being associated with nociceptive mechanisms arising from surgical insult [4].

Pericardial fascial dysfunctions and mediastinal adhesions have been proposed as potential biomechanical and nociceptive contributors to the genesis and perpetuation of pain [1,5]. The continuity of the thoracocervical myofascial system has been described as a possible element involved in the propagation of mechanical tensions towards adjacent regions [7], and mechanotransduction mechanisms have been proposed as a potential biological basis for the transmission of mechanical stimuli through connective tissue [8]. These concepts have also been explored in patients with chronic cervical pain in the absence of prior surgery [7].

The heart is enclosed by the pericardium, a fibroserous sac composed of parietal and visceral layers, which also surrounds the roots of the great vessels and serves protective and supportive functions, limiting cardiac distension [9,10]. The pericardium establishes multiple anatomical relationships with the mediastinum, the sternum, the deep cervical fasciae, and the diaphragm through structures such as the vertebropericardial, sternopericardial, and phrenicopericardial ligaments [10,11]. These connections place the pericardium in anatomical continuity with the fascial structures of the mediastinum, the cervical region, and the diaphragm.

From a neuroanatomical perspective, the pericardium receives innervation from the phrenic nerve, the vagus nerve, and sympathetic fibres arising from the superior cervical and upper thoracic ganglia [10]. This innervation establishes anatomical and neurofunctional continuity between the pericardium and the cervical and thoracic regions, which may help explain the occurrence of referred pain or somatic manifestations in the presence of pericardial alterations, particularly following thoracic surgical procedures [1].

Physiotherapeutic management constitutes an essential component of rehabilitation following cardiac surgery, aimed at promoting postoperative recovery through respiratory physiotherapy and early mobilisation. In patients undergoing coronary artery bypass surgery, various early physiotherapy protocols have been associated with improved autonomic modulation of heart rate and reduced length of hospital stay [12]. However, the specific treatment of pericardial fascial restrictions has been scarcely explored. The medical literature on the pericardium focuses primarily on pericarditis, the management of which is based on non-steroidal anti-inflammatory drugs and colchicine, with corticosteroids reserved for refractory cases. In situations of significant pericardial effusion, cardiac tamponade, or chronic constrictive pericarditis, invasive procedures may be required, including pericardial drainage or pericardiectomy [13].

In contrast, several studies have described the usefulness of osteopathic manipulative treatment (OMT) in the management of fascial and visceral dysfunctions, including the pericardial region [1,2,5], being associated with reductions in pain [1,2,4,5], as well as improvements in respiratory function, which may be associated with enhanced thoracic mobility in the postoperative context [4]. Similarly, in patients with non-specific chronic cervical pain and forward head posture, in the absence of prior surgery, manual therapy has been associated with improvements in craniocervical alignment and postural parameters [7].

The present study does not propose a global osteopathic treatment but rather an intervention specifically focused on the pericardium, with the aim of exploring in isolation its potential influence on chronic post-sternotomy cervicothoracic pain. It is hypothesised that the release of pericardial fascial restrictions may contribute to improved cervical mobility and a reduction in pain symptomatology. This case report, therefore, represents an exploratory approach to this therapeutic strategy.

## Case presentation

A 72-year-old man presented with a history of median sternotomy for triple coronary artery bypass grafting performed on 23 April 2019. Following the surgical intervention, he initially experienced postoperative sternal pain, which progressively resolved over the subsequent months without the need for specific treatment. In the months following surgery, he began to develop progressive cervicothoracic pain accompanied by restricted cervical rotation, which persisted for approximately six years until the initiation of the osteopathic intervention in October 2025. Stiffness and discomfort were more pronounced in the morning, with partial improvement throughout the day during usual daily activity. In the 12 months preceding the osteopathic intervention, no physiotherapeutic or osteopathic treatments targeting his cervicothoracic symptoms were recorded.

The patient had no history of recent trauma or rheumatological disease, and clinical assessment did not reveal neurological deficits or signs of active cervical radiculopathy. No cervical imaging studies were available, and the patient reported that no cervical radiographs or magnetic resonance imaging had been performed prior to the osteopathic intervention. As medical comorbidities, he had arterial hypertension and type 2 diabetes mellitus, and he maintained his usual pharmacological treatment for both conditions throughout the study period, without relevant changes and without reporting the initiation of any new analgesic or anti-inflammatory medication associated with the intervention protocol. The patient was clinically stable and had been evaluated by his cardiologist, with no evidence of active cardiovascular contraindications to the proposed intervention.

The initial physical and osteopathic assessment demonstrated moderate bilateral limitation of cervical rotation, with a slight restriction predominantly towards the right, as well as hypomobility at the T3 and T4 thoracic segments. On palpation, sclerotomal hypersensitivity was noted at C7, T3, and T4, together with increased sensitivity in the sternal dermatome. Osteopathic examination of the cardiac region revealed altered sternal fascial behaviour, with reduced tissue mobility of the anterior thoracic fasciae during the inspiratory phase [14]. This pattern was interpreted as being compatible with a possible fascial restriction.

The study was conducted over three OMT sessions, with an interval of one week between the first and second sessions and two weeks between the second and third sessions. Subsequently, a final evaluation was performed two weeks after the last session, without any additional therapeutic intervention. To facilitate the temporal interpretation of the results, the assessments were organised in a chronological sequence: day 0 (session one), day seven (session two), day 21 (session three), and day 35 (final evaluation, without intervention). Assessments were carried out at multiple time points: immediately before and after each treatment session, as well as at the final evaluation.

Cervical rotation range of motion was measured in the supine position using a digital inclinometer [15]. The device was placed on the vertex of the patient's head and aligned towards the nose to improve measurement reproducibility. To standardise the procedure, the patient was consistently assessed in the same position, and the same verbal instructions were provided, requesting maximal active rotation within the tolerated range without forcing the movement. The final value achieved in each direction was recorded, maintaining the same procedure across all assessments.

Pressure pain threshold was assessed using an analogue algometer, applying pressure over the spinous process of C7. Three consecutive measurements were taken at the same point, with a 30-second interval between them, and the mean of the three measurements was used for analysis [16]. To minimise intersession variability, the patient was consistently assessed in the seated position, and the algometer was applied perpendicular to the bony surface. The anatomical measurement point was identified and kept constant across sessions, being marked to enhance repeatability. Furthermore, pressure was increased progressively and uniformly at an approximate rate of 0.5 kg/cm²/s, maintaining a constant rate of application across all measurements. The patient verbally indicated the moment at which the applied pressure was first perceived as painful, and the corresponding value was recorded as the pressure pain threshold.

Perceived pain was recorded using the numeric pain rating scale (NPRS), on which the patient rated his pain from 0 (no pain) to 10 (maximum imaginable pain) [17]. All measurements and the treatment were performed by the same assessor in order to maintain a consistent procedure throughout the protocol.

OMT focused on the mobilisation and release of the pericardium through four sustained-tension manual techniques directed at the pericardial fasciae and their sternal, cervical, and mediastinal attachment systems. The techniques employed consisted of minor modifications of manual approaches previously described in the literature, adapted and integrated within an osteopathic clinical reasoning framework, sharing conceptual foundations and procedural elements used as methodological references [7]. Each technique was maintained for approximately 120-180 seconds, or until a clear reduction in tissue resistance and increased relative mobility in the direction of restriction were perceived, and was repeated once if a clear limitation persisted. The manual contact was applied progressively through the superficial tissues using minimal pressure, following the perceived tissue resistance. The force remained gentle and low-load throughout the techniques and was adapted continuously to the patient's tissue response, without the application of high-pressure thrust or forceful compression.

During the study period, the patient did not initiate any additional physiotherapeutic or osteopathic treatments, and concomitant therapeutic regimens remained stable, thereby minimising the influence of external factors on the observed outcomes. All techniques described below were applied exclusively to the pericardium and its direct fascial continuities, constituting an intentionally limited protocol rather than a global osteopathic treatment. This methodological choice was made to specifically explore the role of the pericardium in chronic post-sternotomy cervicothoracic pain.

**Figure 1:**
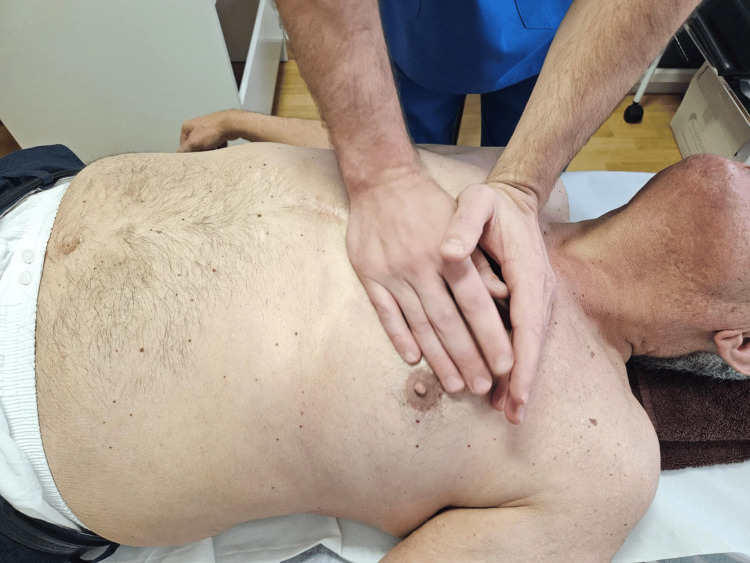
Cardiac axis technique.

Technique for the cardiac axes (Figure 1): Two successive manoeuvres were performed: the short axis (between the pulmonary and tricuspid valves) and the long axis (between the aortic and mitral valves), applying bilateral pisiform contact.

**Figure 2:**
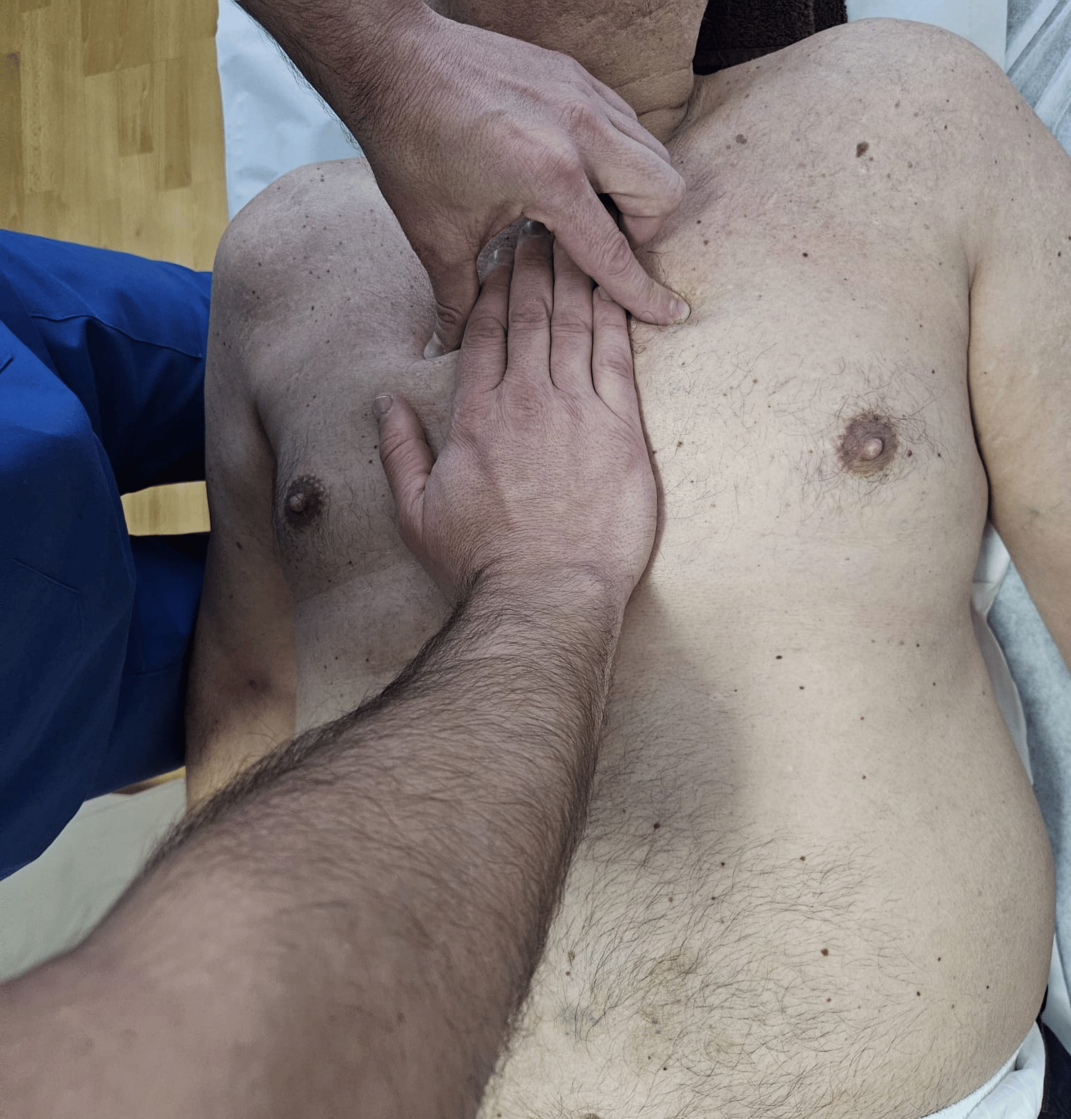
Superior sternopericardial ligament technique.

Technique for the superior sternopericardial ligament (Figure 2): One hand is placed over the sternum, applying tension in a posterior direction (towards the treatment table) and cephalad. The index finger and thumb of the other hand are positioned at the second intercostal space, exerting a pressure vector towards the treatment table and in a caudal direction.

**Figure 3:**
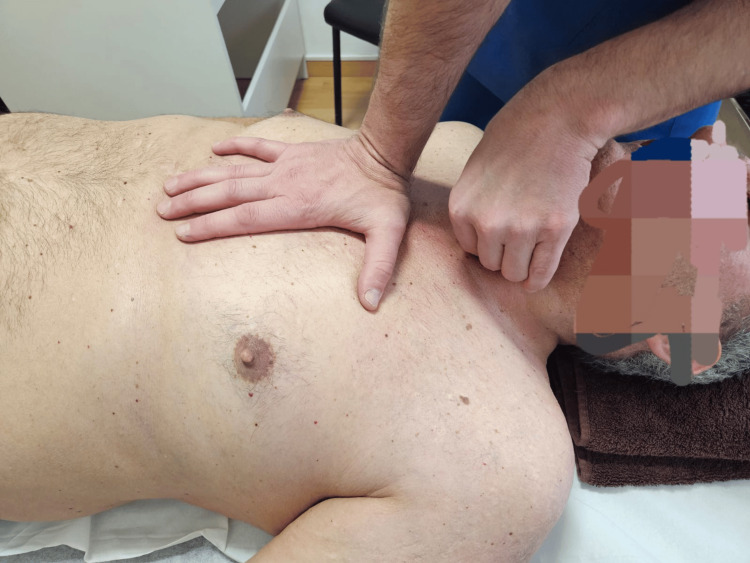
Trachea-pericardium technique.

Trachea-pericardium technique (Figure 3): The cranial hand contacts and stabilises the trachea, following the direction of the restriction (in this case, from left to right). The caudal hand, positioned over the sternal angle, applies sustained tension in a caudal and posterior direction (towards the treatment table) to facilitate the release of mobility restrictions along the fascial continuity.

**Figure 4:**
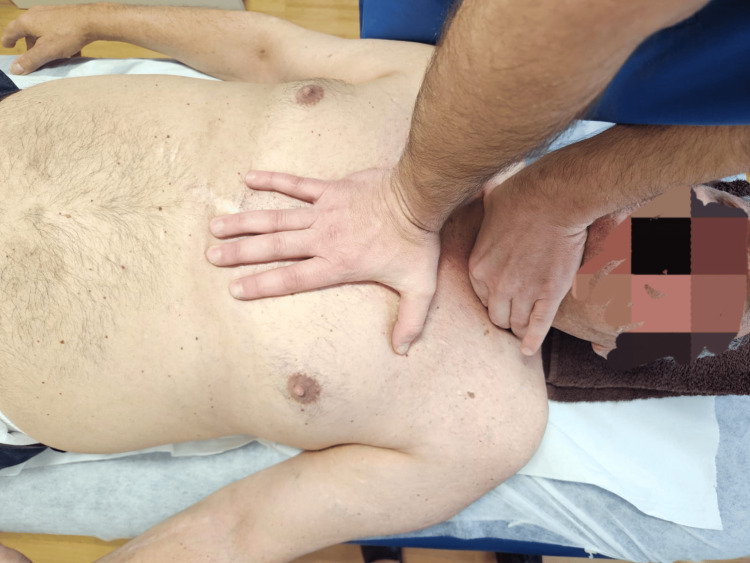
Vertebropericardial ligament technique.

Vertebropericardial ligament technique (Figure 4): The cranial hand contacts and stabilises the anterior tubercles of the transverse processes of C7 using the index finger and thumb. The caudal hand, positioned over the sternal angle, applies sustained tension in a caudal and posterior direction (towards the treatment table) and performs right and left lateral exploration, maintaining the tension in the direction of greatest restriction in order to facilitate the release of restrictions along the vertebropericardial ligaments.

No adverse events or signs of clinical intolerance were recorded during the sessions. The protocol, described step by step, can be reproduced by other therapists experienced in OMT by following the manoeuvres and contact points detailed above.

Results

Cervical mobility, assessed using a digital inclinometer, demonstrated progressive improvement throughout the course of treatment (Table 1). Right cervical rotation increased from 34° to 54° and left rotation from 38° to 55°. These values approached ranges described as functional in the older adult population.

**Table 1:** Cervical rotation range of motion measured by a digital inclinometer.

Assessment time point	Right rotation (°)	Left rotation (°)
Day 0 - Pre-session 1	34	38
Day 0 - Post-session 1	40	43
Day 7 - Pre-session 2	39	44
Day 7 - Post-session 2	45	49
Day 21 - Pre-session 3	46	49
Day 21 - Post-session 3	55	55
Day 35 - Final evaluation	54	55

Pressure pain threshold over the spinous process of C7 increased progressively from 1.2 kg/cm² to 2.8 kg/cm² at the final evaluation (Table 2).

**Table 2:** Pressure pain threshold measured over the spinous process of C7.

Assessment time point	Pressure pain threshold (kg/cm²)
Day 0 - Pre-session 1	1.2
Day 0 - Post-session 1	1.6
Day 7 - Pre-session 2	1.8
Day 7 - Post-session 2	2.2
Day 21 - Pre-session 3	2.1
Day 21 - Post-session 3	2.6
Day 35 - Final evaluation	2.8

In the assessment of pain using the NPRS, the patient recorded an initial score of 7/10 (Table 3). The values obtained immediately after each session were similar, likely due to transient discomfort associated with pericardial manipulation. Nevertheless, the patient reported a perceptible reduction in pain within 24 hours following each session. At the final evaluation, the score had decreased to 2/10. Additionally, a telephone follow-up was conducted on day 67 (32 days after the final evaluation), during which the patient reported maintenance of the clinical improvement, with an NPRS score of 2/10, without the occurrence of adverse events or new symptoms. This follow-up assessment was subjective in nature and did not include further objective measurements (digital inclinometry or algometry).

**Table 3:** Pain intensity measured using the numeric pain rating scale (NPRS).

Assessment time point	NPRS
Day 0 - Pre-session 1	7
Day 0 - Post-session 1	7
Day 7 - Pre-session 2	5
Day 7 - Post-session 2	5
Day 21 - Pre-session 3	3
Day 21 - Post-session 3	3
Day 35 - Final evaluation	2
Day 67 - Telephone follow-up	2

An increase in cervical mobility, a reduction in pain intensity, and an elevation in pressure pain threshold were observed, and these improvements were maintained for at least approximately two months from the initiation of the protocol.

## Discussion

The findings of this clinical case suggest that a therapeutic approach exclusively directed at the pericardium may be associated with objective improvements in cervical mobility, increased pressure pain threshold, and reduced perception of chronic cervicothoracic pain following median sternotomy. It should be emphasised that the observed improvements occurred in the absence of direct intervention on the cervical spine, thoracic spine, diaphragm, or other musculoskeletal structures typically included in a global osteopathic treatment approach.

Importantly, the reduction in pain intensity observed in this case exceeded the minimal clinically important difference (MCID) for the NPRS, which has been reported to be approximately 2 points in patients with musculoskeletal pain. The improvement of 5 points observed in this patient, therefore, represents a clinically meaningful change, supporting the potential relevance of the intervention from the patient's perspective.

From an anatomical perspective, the pericardium presents direct ligamentous attachments to the sternum, diaphragm, and vertebral column, as well as close mediastinal fascial continuities with vascular and tracheobronchial structures, including the tracheobronchopericardial ligaments [10,14]. It has been suggested that, through these ligamentous connections, the pericardium may influence cervical posture and be associated with cervical pain, even in the absence of prior surgical intervention [7]. In the context of cardiac surgery, persistent pain sequelae have been described, including post-sternotomy pain syndromes and other forms of chronic postoperative thoracic pain [5]. The pathophysiology of these conditions may involve mediastinal adhesion phenomena and alterations of pericardial tissue [1,5].

On the basis of these considerations, potential structural or mechanical modifications of the pericardium could alter its behaviour within the thoracic complex and the relative mobility of the involved tissue planes. Considering the continuity of the fascial system described in the literature, such alterations could theoretically contribute to cervicothoracic mechanical imbalances and functional repercussions both in adjacent regions and at a distance [8]. In this patient, the persistence of pain and the limitation in cervical mobility suggest that these mechanisms may have contributed to the chronicity of the clinical presentation; however, this interpretation should be understood as a plausible clinical association rather than as direct mechanistic evidence.

It cannot be ruled out that the cervicothoracic symptomatology may have been influenced by age-related degenerative changes or by other concomitant musculoskeletal factors, as well as by the natural variability of chronic pain and patient expectations. However, the stability of the condition during the years preceding treatment and the improvement observed following its initiation suggest a possible temporal relationship between the intervention and the clinical course.

In addition to the biomechanical mechanisms previously outlined, the innervation of the pericardium is complex and includes phrenic, sympathetic, and vagal afferents [10,11]. The phrenic nerve runs in close proximity to the pericardium along its mediastinal course and gives off sensory branches to the pericardial sac [18], providing the anatomical basis for somatic pain. Sympathetic afferents participate in reflex mechanisms of segmental integration, whereas vagal fibres are primarily involved in cardiac autonomic regulation [10,11]. Accordingly, osteopathic interventions directed at the pericardium may contribute to the improvement of pain symptomatology, as has been described in cardiothoracic and postoperative contexts [1,5].

It may be hypothesised that the persistence of atypical afferent signalling originating from deep thoracic structures, in the absence of evident structural pathology, could act as a contributing factor in processes of central sensitisation, characterised by amplification of pain perception and altered pain thresholds. This mechanism may help explain the persistence of chronic pain in this patient [19]. In this context, osteopathic manual therapy might be associated with changes in central pain processing and autonomic regulation rather than with direct local effects, potentially contributing to the observed reduction in pain experience and functional improvement. However, the current evidence regarding the neurophysiological mechanisms by which manual therapy influences central processes involved in pain modulation remains limited and controversial [20]. Therefore, this interpretation should be regarded as a plausible theoretical explanation rather than as a demonstrated causal relationship.

Overall, the findings of the present case are consistent with the available evidence, derived primarily from case reports and one randomised controlled trial, which have described favourable outcomes in respiratory function, cervicothoracic mobility, and pain reduction in cardiothoracic and postoperative contexts [1,2,4,5]. However, the methodological heterogeneity and the inherent risk of bias associated with such study designs limit the strength of the conclusions that can be drawn.

As is customary in a single case report, this study presents limitations inherent to its design, including the absence of a control group, lack of assessor blinding, and potential variability in objective measurements (algometry) and subjective scales (NPRS) [16,17], despite efforts to standardise the measurement procedures. In addition, the same practitioner performed both the osteopathic intervention and the outcome assessments, which may have introduced assessor bias and should be considered when interpreting the results. Furthermore, the telephone follow-up conducted on day 67 from the initiation of the protocol was exclusively subjective and did not include objective reassessment, thereby limiting the interpretation of the medium-term maintenance of the observed outcomes. Evidence regarding pericardial OMT following sternotomy remains limited and is predominantly based on observational designs; therefore, the results should be interpreted with caution. The exclusive application of treatment to the pericardium represents both a methodological limitation and an exploratory strength, as it allowed preliminary investigation of the contribution of this focused approach without the influence of a global osteopathic intervention.

Nevertheless, this case suggests that the inclusion of pericardial OMT as an adjunctive therapeutic strategy may represent a potentially useful approach within the context of post-sternotomy rehabilitation, contributing to improvements in cervical mobility and reductions in pain symptomatology, particularly when integrated within an individualised treatment framework targeting mediastinal fascial restrictions. Future research should include controlled trials with larger sample sizes, standardised assessments of thoracic function, longitudinal designs with long-term follow-up, and evaluation of functional impact on activities of daily living, as well as investigation of the neurophysiological and myofascial effects associated with OMT, in order to clarify the underlying physiological mechanisms and more precisely determine its clinical efficacy.

## Conclusions

This clinical case suggests that an OMT protocol exclusively directed at the pericardium may be associated with clinically relevant changes in a patient with a history of median sternotomy and chronic cervicothoracic pain. Following three treatment sessions, improvements were observed in cervical rotation, pressure pain threshold over C7, and perceived pain intensity. These findings support the hypothesis that pericardial fascial restrictions may contribute to post-sternotomy cervicothoracic symptomatology, potentially through biomechanical and neurophysiological mechanisms.

Although this is a single case and the findings must be interpreted with caution, this study suggests that pericardial-focused OMT may represent a potentially valuable adjunct within post-sternotomy rehabilitation, particularly when the aim is to explore in isolation the possible contribution of the pericardium without the influence of a global osteopathic intervention. The absence of observed adverse events supports the feasibility of this approach in clinically stable patients. Controlled studies with larger sample sizes, standardised assessments, long-term follow-up, and assessor blinding are required to confirm these findings and to clarify the underlying mechanisms involved, including outcome measures related to functional limitation and quality of life.
